# Familial Acromegaly and Bilateral Asynchronous Pheochromocytomas in a Female Patient With a *MAX* Mutation: A Case Report

**DOI:** 10.3389/fendo.2021.683492

**Published:** 2021-05-31

**Authors:** Elizaveta Mamedova, Evgeny Vasilyev, Vasily Petrov, Svetlana Buryakina, Anatoly Tiulpakov, Zhanna Belaya

**Affiliations:** ^1^ Department of Neuroendocrinology and Bone Diseases, Endocrinology Research Centre, Moscow, Russia; ^2^ Department and Laboratory of Inherited Endocrine Disorders, Endocrinology Research Centre, Moscow, Russia; ^3^ Department of Radiology, Endocrinology Research Centre, Moscow, Russia; ^4^ Department of Endocrine Genetics, Research Centre for Medical Genetics, Moscow, Russia

**Keywords:** acromegaly, pheochromocytoma, familial pituitary adenomas, MAX, case report

## Abstract

**Background:**

There are very few cases of co-occurring pituitary adenoma (PA) and pheochromocytomas (PCC)/paragangliomas caused by *MAX* mutations. No cases of familial PA in patients with *MAX* mutations have been described to date.

**Case Presentation:**

We describe a 38-year-old female patient, presenting with clinical and biochemical features of acromegaly and PCC of the left adrenal gland. Whole-exome sequencing was performed [NextSeq550 (Illumina, San Diego, CA, USA)] identifying a nonsense mutation in the *MAX* gene (NM_002382) [c.223C>T (p.R75X)]. The patient had a medical history of PCC of the right adrenal gland diagnosed aged 21 years and prolactinoma diagnosed aged 25 years. Cabergoline treatment was effective in achieving remission of prolactinoma at age 33 years. The patient’s father who died at age 56 years of a heart attack had a medical history of PA and prominent acromegalic features, which supports the familial presentation of the disease.

**Conclusion:**

This clinical case gives an insight into the clinical presentation of familial PA and PCC probably associated with a *MAX* mutation.

## Introduction

In 2015, Xekouki et al. suggested the term “3 PAs” to denote a combination of pituitary adenomas (PAs) and pheochromocytomas (PCCs) and paragangliomas (PGLs) (1). It was shown that mutations in genes coding succinate dehydrogenase subunits (*SDHx*) can be identified in up to 75% of cases of “3 PAs” in the presence of a family history (1). In the same year O’Toole et al. summarized the data on 72 published cases with such a combination (2). Twenty nine percent of these patients harbored mutations in the genes responsible for the development of hereditary PCC/PGL or PA (*MEN1, RET, SDHB, SDHC, SDHD, SDHAF2, SDHA + VHL*), 32% had suspicious family history, and 39% were index cases without identified mutations (2). No more than 100 cases of “3 PAs” have been reported to date (3).

In 2011, it was shown that Myc-associated factor X (*MAX*) gene mutations predispose to the development of hereditary PCC/PGL (4). Since 2012, several cases of co-occurrence of PA and PCC/PGL due to *MAX* mutations have been reported (5–9).

The rarity of the co-occurrence of PA and PCC/PGL and a very limited number of described cases of such a combination caused by *MAX* mutations make the description of each novel case of particular importance. We describe a clinical case of familial acromegaly in a patient with a *MAX* mutation.

## Methods

Whole body computed tomography (CT) was performed on Revolution CT (GE, Milwaukee, WI, USA). Magnetic-resonance imaging (MRI) was performed on Signa Pioneer 3.0 T (GE, Milwaukee, WI, USA).

Genomic DNA from peripheral blood lymphocytes was isolated using the PureLink Genomic DNA Mini Kit according to the manufacturer’s protocol (Thermo Scientific, Waltham, MA, USA). Ultrasonic DNA fragmentation was performed on a Covaris S220 device (Covaris Inc., Woburn, MA, USA). Sample preparation for sequencing libraries and exome enrichment were performed using the TruSeq DNA Exome kit according to the manufacturer’s instructions (Illumina, San Diego, CA, USA). Pair-end reading of the obtained libraries (2 × 80 bp) was carried out on a NextSeq550 sequencer (Illumina, San Diego, CA, USA). Bioinformatics analysis was carried out using Genome Analysis ToolKit (GATK) ver. 4.1.2.0 (Broad Institute, Cambridge, MA, USA) and ANNOVAR ver. 2018Apr16 (10) software packages.

## Case Description

A 38-year-old female patient was admitted to our hospital with high blood pressure up to 180/100 mm Hg with complaints of fatigue and high blood pressure over the last year.

Her medical history showed similar symptoms at the age of 21 years, when she was hospitalized due to high blood pressure (180/100 mm Hg), dizziness, and weakness. There was an increase of vanillylmandelic acid in daily urine up to 19.7 mg/day (0–7). Computed tomography (CT) revealed a tumor of the right adrenal gland 6.1 × 4.4 × 4.2 cm. A right-side adrenalectomy was performed, leading to the normalization of blood pressure, and a diagnosis of PCC was histologically confirmed. At the age of 25, due to the development of secondary amenorrhea and galactorrhea, she was re-examined, showing high prolactin (PRL) levels >7.000 mIU/L (64–395). Magnetic resonance imaging (MRI) revealed a PA 14 × 24 × 17 mm. PRL levels normalized with cabergoline treatment (1.5 mg per week). Six months after cabergoline prescription, PA decreased to 12 × 21 × 16 mm. At the age of 28 years, the patient became pregnant, so cabergoline was temporarily discontinued. Subsequently at the age of 33 years, due to repeated prolactin levels within the reference range, cabergoline was discontinued.

**Figure 1:**
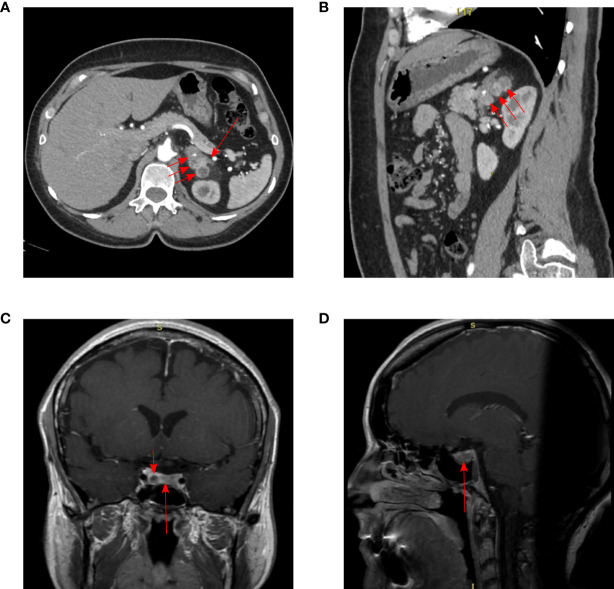
Imaging results in our patient. CT of the abdomen: **(A)** arterial phase, axial projection, **(B)** arterial phase, sagittal projection. Three pheochromocytomas in the body and medial pedicle of the left adrenal gland (short arrows). Uniformly thickened lateral pedicle of the left adrenal gland (long arrow). The adrenal lesions were located close to each other, 21 × 22 × 23 mm, 24 × 14 × 19 mm and 24 × 22 × 24 mm. MRI of pituitary adenoma (long arrow) with cystic component (short arrow), subtotally replaces adenohypophysis: **(C)** T1 CE (contrast-enhanced) coronal projection, **(D)** T1 CE sagittal projection. The size of pituitary adenoma was 22 × 8 × 14.8 mm.

**Figure 2:**
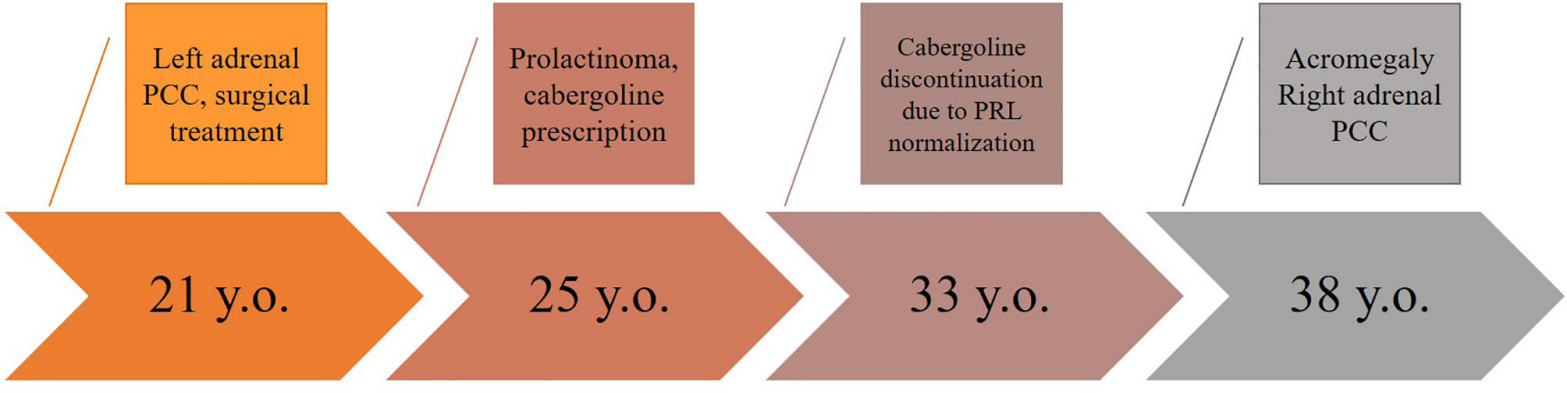
Timeline of disease progression.

At the time of admission at the age of 38 years, acromegaloid facial features (bulbous nose, prognatism), enlarged hands, and feet were noted. The patient reported that her father had a similar appearance, which was confirmed by family photographs. Based on the available medical information, the patient’s father received radiation therapy for PA when he was a young adult and died from a heart attack at the age of 56. Laboratory data of the patient confirmed acromegaly based on increased insulin-like growth factor 1 (IGF-1)—451.4 ng/ml (82–283), nadir GH during oral glucose tolerance test >1 ng/ml, moderate increase in PRL levels 489.1 ng/ml (64–395). MRI showed a pituitary macroadenoma (**Figure 1**). PCC was diagnosed based on hypertension, urinary metanephrine 665.7 µg/24 h (25–312), normetanephrine 1619.1 µg/24 h (35–445) levels. Abdominal CT showed three round soft tissue lesions in the left adrenal gland (**Figure 1**). Timeline of the disease progression is shown in **Figure 2**.

Given the development of two endocrine tumors and a positive family history in our patient, whole-exome sequencing was performed. A germline heterozygous nonsense mutation in exon 4 of the *MAX* gene (NM_002382: c.223C>T (p.Arg75X)) was revealed (allele frequency was not found in GnomAD (https://gnomad.broadinstitute.org) and is 0.00000795 according to VarSome (https://varsome.com/variant/hg19/14-65544703-G-A?annotation-mode=germline). This variant is classified as pathogenic, with five pathogenic predictions by *in silico* algorithms (BayesDel_addAF, DANN, EIGEN, FATHMM-MKL, and MutationTaster) and has no benign predictions. A variant of uncertain significance in the *RET* gene was also found (NM_020975: c.1915G>A: p.Ala639Thr), allele frequency 0.00003 (https://gnomad.broadinstitute.org). This variant was predicted as pathogenic by three *in silico* algorithms (FATHMM-MKL, M-CAP, MutationTaster) and as benign by nine *in silico* algorithms (BayesDel_addAF, DANN, DEOGEN2, EIGEN, LIST-S2, MVP, MutationAssessor, PrimatteAI, SIFT). No pathogenic variants in *AIP*, *MEN1*, or *SDHx* genes were found.

At the time of admission, the patient declined surgical treatment of PCC; therefore, our multidisciplinary team decided that it was not safe to perform transsphenoidal surgery as the first step. Therapy with doxazosin was prescribed to normalize blood pressure due to PCC, and the dosage was titrated to 5 mg/day, after which her blood pressure stabilized. Long-acting somatostatin analogs were prescribed for acromegaly, but her IGF-1 remained elevated [424.7 ng/ml (82–283)] on long-acting octreotide 20 mg/28 days three months after prescription. Thus, the dosage was elevated to 30 mg/28 days; control IGF-1 has not been checked yet.

## Discussion

In 2012, Bournichon et al. described a cohort of patients with *MAX* mutations and PCC/PGL, where a PA in a male patient was mentioned (without further description of the details), and it was shown that other tumors can occur in mutation carriers (breast cancer, renal oncocytoma, squamous cell carcinoma of the tongue, renal carcinoma) (5). In 2017, Roszko et al. described a case of a combination of prolactinoma and bilateral PCC (6), and in 2018, Daly et al. described another three cases of a combination of PA and PCC due to *MAX* mutations (7). One further case was described by Kobza et al. in 2018 (8). In 2020, Seabrook et al. described two families with *MAX* mutations which were remarkable not only for the presence of PA and PCC/PGL (metastatic in some cases), but also for the presence of many other tumors (paravertebral ganglioneuroma, abdominal neuroblastoma, multiple parathyroid adenomas, chondrosarcoma, multifocal pulmonary adenocarcinomas), suggesting *MAX* as a novel multiple endocrine neoplasia gene (9).

The distinctive feature of this case is that our patient had a positive family history of PA. Based on the available documentation, the patient’s father had prominent acromegalic features and received radiotherapy due to PA. Because neither the patient’s PA nor PCC tissues were available for MAX immunohistochemical (IHC) analysis due to her refusal to undergo surgery and because we did not have her father’s DNA to perform genetic testing, it is not completely evident that a *MAX* mutation is responsible for familial acromegaly in this case. Nevertheless, taking into account the obvious presence of familial acromegaly in our case (the recorded medical history of radiation therapy due to PA and prominent acromegalic features of the patient’s father), apparent paternal transmission of the disease (4), and the identification of a pathogenic *MAX* mutation, we could hypothesize that the incidental co-occurrence of familial acromegaly of another unidentified etiology and PCC due to a *MAX* mutation in one case is highly unlikely.

The mutation identified in our case was previously described in bilateral PCC (4, 11, 12), as well as in an 18-year-old man with bilateral PCC and primary hyperparathyroidism (5), supporting the likelihood of this being a true pathogenic mutation.

The clinical features of patients with a combination of PA and PCC due to mutations in the *MAX* gene described in the literature (5–9) are summarized in **Table 1**. As can be seen from the table, the disease can occur in both men and women, and it presents with both macro- or microPA. It is noteworthy that only prolactinomas (micro- and macroadenomas) and somatotropinomas (macroadenomas) have been described. Generally, prolactinomas successfully responded to medical treatment, while somatotropinomas required a multimodal approach to achieve remission. Most patients had bilateral PCC, which were synchronous or asynchronous (sometimes multiple). In a recent work by Seabrook et al. two additional cases suspicious for the combination of PA and PCC/PGL have been described. In one family, there was a patient with acromegaly, but it was not clear whether it was due to a PA or a growth hormone-releasing hormone-secreting PGL and another patient with pituitary enlargement with intermittently mildly elevated serum IGF-1 and normal GH suppression and no clinical features of acromegaly. In the other family, there was a patient with a microprolactinoma (not requiring dopamine agonist therapy) (9).

Table 1Clinical features of patients with “3 PAs” and *MAX* mutations.

**Table:** 

Case, reference			Bournichon et al. (5)	Roszko et al. (6)	Daly et al. (7)			
					1	2	3	
Gender			M	F	M	F	M	
Manifestation			NA	PA	PCC	PA	PA	
Age of manifestation, years			57	35	30	26	16	
PA		Type of secretion	NA	Prolactinoma	Prolactinoma	Somatotropinoma	Somatotropinoma	
		PA size	NA	Macro	Micro	Macro	Macro	
		Treatment	NA	Cabergoline, medical subcompensation	Cabergoline, medical remission	Somatostatin analogs, cabergoline, pegvisomant, medical remission	Surgical treatment, radiation therapy, remission	
PCC		Unilateral/bilateral	Unilateral?	Bilateral, synchronous	Unilateral (relapse)	Bilateral, synchronous	Bilateral, asynchronous (relapse)	
		Solitary/multiple	Multiple	two nodules (left adrenal), one nodule (right adrenal)	one nodule in the right adrenal, later—relapse (one nodule in the position of right adrenal)	1one nodule in the left adrenal, one nodule in the right adrenal	one nodule in the left adrenal, later—one nodule in the right adrenal, one nodule in the position of the left adrenal (relapse)	
		Prevailing type of secretion	NA	Normetanephrine	NA	NA	NA	
Family history			No	No	No*	No	No	
*MAX* mutation			c.220A>G p.Met74Val	c.296-1G>T	Exon 3 deletion	Exons 1–3 and intron 3 deletions	Exon 4 deletion	
IHC (MAX staining) and genetic analysis (LOH) of tumors			PCC: IHC—positive; LOH—detected	PCC: IHC—negative	PCC: IHC—negative	PCC: IHC—negative; LOH—detected Thyroid carcinoma: IHC—positive	PCC: IHC—negative

**Table:** 

Case, reference		Kobza et al. **(****8****)**	Seabrook et al. **(****9****)**				Our case
			**F1 II.1**		**F1 III.5**	**F2 II.1**	
Gender		F	M		F	F	F
Manifestation		PA	PCC		PCC	PCC	PCC
Age of manifestation, years		33	20		14	21	21
PA	Type of secretion	Prolactinoma	Somatotropinoma? Growth hormone-releasing hormone producing PGL?		No PA (pituitary enlargement)?, “intermittently elevated IGF-1 levels, normal GH suppression on OGTT, no symptoms or signs of GH excess”.	Prolactinoma (or non-functioning)?	Prolactinoma/Somatotropinoma
	PA size	Micro	NA		–	Micro	Macro
	Treatment	Cabergoline, medical remission	NA		–	No treatment	Cabergoline, medical remission, Somatostatin analogs
PCC	Unilateral/bilateral	Bilateral, synchronous	Right PC, left ‘para-aortic’ PGL		Bilateral, asynchronous	Bilateral, synchronous (relapse at age 64, metastatic)	Bilateral, asynchronous
	Solitary/multiple	two nodules (left adrenal), one nodule (right adrenal)	NA		NA	NA	3 nodules (left adrenal), right adrenal - NA
	Prevailing type of secretion	Normetanephrine	NA		Normetanephrine	NA	Normetanephrine
Family history		No	Yes		Yes	Yes (multiple tumors)	Yes (PA)
*MAX* mutation		c.171+2T>A	c.200C>A p.Ala67Asp		c.200C>A p.Ala67Asp	c.22G>T p.Glu8X	c.223C>T p.R75X
IHC (MAX staining) and genetic analysis (LOH) of tumors		Not performed	PCC/ganglioneuroma: IHC—negative		PCC: IHC—negative	NA	Not performed

f, female; m, male; macro, macroadenoma, micro, microadenoma; NA, no data available; IHC, immunohistochemical analysis; LOH, loss of heterozygosity. *The son of the patient had neuroendocrine tumor of the pancreas without other endocrine manifestations. F1, family 1; F2, family 2 (9).

A wide range of other tumors has been described in patients with *MAX* mutations: breast cancer, renal oncocytoma, squamous cell carcinoma of the tongue, renal carcinoma (5), ganglioneuroblastoma, ganglioneuroma, chondrosarcoma, lung adenocarcinoma, parathyroid adenomas (9, 13). In patients with a combination of PA and PCC and *MAX* mutations (**Table 1**), a follicular variant of papillary thyroid cancer was described in case 2 (7), and in the son of case 1 (7) with the same confirmed mutation—deletion of exon 3—a neuroendocrine tumor of the pancreas was detected in the absence of other manifestations (14), as well as a rib chondrosarcoma and multiple parathyroid tumors in a case described by Seabrook et al. (9); thus it can be assumed that for *MAX* mutation carriers, life-long surveillance in order to detect early various tumors may be necessary.

The *MAX* gene encodes a Myc-associated factor X, which is a leucine zipper type transcription factor and a member of the MYC/MAX/MSD network of proteins involved in cell proliferation, differentiation, and apoptosis (4). Mutations in the *MAX* gene predispose to the development of abdominal PCC/PGL of the normetanephrine type of secretion, with bilateral lesions (synchronous and asynchronous) in 50% of cases and 40% of cases having a positive family history (15). When conducting an IHC study of the removed PCC in some of the “3 PA” described cases (**Table 1**), the absence of nuclear expression of MAX was revealed (6, 7, 9). MAX expression was also absent in the pancreatic neuroendocrine tumor of the son of patient 1 (7, 14), while its expression was retained in the thyroid cancer tissue (7). The detection of LOH in some tumors supports the role of *MAX* as a tumor-suppressor gene (5, 7). PA tissue in all the cases was not available for analysis, so the role of *MAX* gene mutations in the pathogenesis of PA remains unclear.

## Conclusion

In summary, this clinical case gives an insight into the clinical presentation of familial PA and PCC probably associated with a *MAX* mutation. The mechanisms of co-occurrence of PA and PCC/PGL are still poorly understood.

## Data Availability Statement

The original contributions presented in the study are included in the article/supplementary material. Further inquiries can be directed to the corresponding author.

## Ethics Statement

The studies involving human participants were reviewed and approved by the local ethics committee of the Endocrinology Research Center. The patients/participants provided their written informed consent to participate in this study.

## Author Contributions

All authors contributed to the study conception and design. Literature search, writing of the article, and the patient’s attending physician: EM. NGS and data analysis: EV, VP, and AT. CT and MRI performance and interpretation: SB. Final editing of the article: AT and ZB. All authors contributed to the article and approved the submitted version.

## Funding

This work was supported by the Grant of the President of the Russian Federation for young investigators МК-1100.2020.7

## Conflict of Interest

The authors declare that the research was conducted in the absence of any commercial or financial relationships that could be construed as a potential conflict of interest.
